# Investigating Development and Defense Systems in Early Reproductive Stages of Male and Female Gonads in Black Scorpionfish *Scorpaena porcus* (Linnaeus, 1758)

**DOI:** 10.3390/biology13080587

**Published:** 2024-08-02

**Authors:** Alessio Alesci, Sebastian Marino, Claudio D’Iglio, Silvana Morgante, Anthea Miller, Gabriele Rigano, Josipa Ferri, Jorge M. O. Fernandes, Gioele Capillo

**Affiliations:** 1 Department of Chemical, Biological, Pharmaceutical and Environmental Sciences, University of Messina, 98166 Messina, Italy; claudio.diglio@unime.it (C.D.); silvanamorgante@virgilio.it (S.M.); gabrielerigano99@gmail.com (G.R.); gcapillo@unime.it (G.C.); 2 Department of Veterinary Sciences, University of Messina, 98166 Messina, Italy; anthea.miller@studenti.unime.it; 3 University Department of Marine Studies, University of Split, 21000 Split, Croatia; jferri@more.unist.hr; 4 Institute of Marine Sciences, CSIC, 08003 Barcelona, Spain; 5 Sea in Health and Life SRL., c/o Department of Chemical, Biological, Pharmaceutical and Environmental Sciences, 98164 Messina, Italy

**Keywords:** *Scorpaena porcus*, teleost, internal defense system, fish reproduction, Piscidin-1

## Abstract

**Simple Summary:**

The present study investigates the reproductive and immune defense systems in the gonads of black scorpionfish (*Scorpaena porcus*, Linnaeus 1758). This study concentrates on the initial phases of reproduction in both male and female fish and investigates the presence and function of an antimicrobial peptide, Piscidin-1. Histological analyses demonstrate morphological changes from the immature to developing stages in gonads. Immunohistochemical methods demonstrate strong reactivity to Piscidin-1 in germinal and somatic cells during early ontogeny, suggesting its crucial role in local defense mechanisms. This research contributes to the understanding of the interaction between reproductive biology and immune defense in *S. porcus*, offering insights that could benefit species conservation and management efforts.

**Abstract:**

One of the most crucial biological indicators in tracking long-term variations in the reproductive cycle is sexual development. *Scorpaena porcus* (Linnaeus, 1758), commonly known as the black scorpionfish, is a small teleost from the family Scorpaenidae. Much is known about its ecology, but data on its reproductive and defense systems are still lacking. Antimicrobial peptides (AMPs), such as piscidins, are integral components of the innate immune system in fish. These peptides exhibit a wide range of activity against bacteria, fungi, viruses, and protozoa and act as the first line of host defense. This study aims to investigate the primary sexual development stages in male and female gonads of black scorpionfish, providing additional knowledge on the reproductive biology of this teleost while evaluating concomitant changes in the expression of a Piscidin-1 antimicrobial peptide. The results show a histological, morpho-structural change from the immature stage to the developing virgin stage. Immunohistochemical analyses show that germinal and somatic cells are strongly reactive to Piscidin-1 in both gonads at an early ontogeny stage. These data suggest that Piscidin-1 may play a key role in the local defense system of scorpionfish gonads at this delicate stage, which is critical for the continuation and maintenance of the species. The present findings are potentially useful for a better understanding of the reproductive cycle of this fish, improving our knowledge of the interaction between the immune system and reproduction.

## 1. Introduction

Sexual development status is a crucial biological parameter for determining the spawning season of a species and monitoring long-term changes in the reproductive cycle [1]. However, in the Mediterranean Sea, fundamental biological information on many teleost species and the knowledge base on reproduction and its associated defense system is still lacking [2].

In vertebrates, the gonad is regarded as an immunologically privileged site where immune responses act differently [3]. Only recently, the regulation of immune function within teleost’s reproductive organs and the implications of pathogen dissemination in the gonads have been documented [4]. Antimicrobial peptides (AMPs) are critical, first-line defenses against many pathogens and have been extensively studied in invertebrates and vertebrates, teleost fish included [5,6]. Their low molecular weight, polarity, and amino acid composition give them a broad spectrum of antimicrobial activities against bacteria, viruses, fungi, protozoa, and even cancer cells [7]. In teleost fish, over 60 AMPs have been described, and their expression has been determined in various tissues, including the gonads [8,9,10]. However, it is important to study their role in the reproductive organs as well since the regulation of the immune response in these organs differs [11].

Piscidins are a subgroup of amphipathic polypeptides that range from 18 to 46 amino acid residues. They are among the most potent AMPs found in both freshwater and marine teleosts and are able to inhibit the growth of bacteria, fungi, viruses, and parasites at submicromolar concentrations [12]. Several immunohistochemical studies have demonstrated that different cell types in various tissues and organs, particularly mucosal tissues mediating contact with the environment (such as gills, skin, and the alimentary tract) and hematopoietic tissues, are involved in the production of piscidin [13,14,15,16,17,18,19,20,21,22].

*Scorpaena porcus* (Linnaeus, 1758) is a benthic species widely distributed in the Eastern Atlantic, the Mediterranean, and the Black Sea [23]. It inhabits shallow rocky bottoms or mixed rocky–sandy seabed and is also able to extend its bathymetric distribution until the bathyal plan up to a depth of 800 m. This species commonly dwells among crevices and rocks or on seagrass beds [24,25]. It is a benthic predator, feeding mainly on crustaceans and small fishes, and is also an occasional form of prey for other predators; it is an essential part of the trophic food webs of rocky reef ecosystems in both temperate and tropical waters [26]. In the western and eastern parts of the Mediterranean Sea, *S. porcus* is one of the main target species of artisanal fisheries, with relevant catch-decline and size-composition shifts reported in several areas, mainly related to the high fishing pressure [27,28,29,30,31]. In the North-Western Mediterranean Sea, it is typically found at depths between 10 and 30 m and rarely below 80–90 m, while in the Black Sea, it is found at depths between 10 and 30 m and rarely beyond 50 m. Even though *S. porcus* is widely distributed and has a significant economic impact, certain features of its biology, such as its defense system and reproduction, are not yet well understood [32]. From a reproductive perspective, the family Scorpaenidae is particularly interesting because it includes species that exhibit a variety of reproductive strategies, from simple oviparity to matrotrophic viviparity, with the eggs embedded in a gelatinous matrix [33].

This study aims to assess morpho-structural changes in the gonads of *S. porcus* during the early reproductive stages while also analyzing, for the first time in this species, the expression of an immune-related antimicrobial peptide (Piscidin-1) in the reproductive organs.

## 2. Materials and Methods

### 2.1. Specimen Collection and Sample Processing

*S. porcus* specimens (N = 40; 20 per sex; and 10 per maturity stage) were caught manually by local fishermen using a sampling net from the rocky bench present on the beachfront between March and April 2023, thanks to the limited depth (ranging from 0.2 to 1.2 m) of the tidal ponds in the “beach rock” formations toward the Sicilian coast of the Strait of Messina. Each individual specimen was anesthetized with an overdose of tricaine methanesulfonate (MS 222; 0.5 g L^−1^), measured (total length, TL, mm), and weighed (total weight, TW, g). Then, samples of male and female gonads were taken and classified macroscopically, according to Follesa and Carbonara [2], into M1 (immature testis), M2 (developing virgin testis), F1 (immature ovary), and F2 (developing virgin ovary) (Table 1), and then processed following the standard procedures used to create durable preparations for optical microscopy and paraffin block storage.

### 2.2. Tissue Preparation for Histology and Histochemistry

The samples from 40 specimens were dehydrated using graded ethanol after being fixed for 15 to 20 h in a 4% paraformaldehyde solution in 0.1 M phosphate-buffered saline (pH 7.4). They were then washed in xylene. After that, samples were embedded in Paraplast^®^ (McCormick Scientific LLC, St. Louis, MO, USA), and a rotary microtome (LEICA 2065 Supercut, Nussloch, Germany) was used to cut serial slices that were 3–5 μm thick. The slices were stained with Mallory trichrome (04-020802 Bio-Optica Milano S.p.A., Milan, Italy) and May–Grünwald Giemsa (04-081802 Bio-Optica Milano S.p.A., Milan, Italy) in order to examine them under a light microscope [34].

### 2.3. Identification of Piscidin-1 Ortholog Protein in Pterois Miles Through In Silico Prediction

To confirm the presence of Piscidin-1 in teleost fish, *Pterois miles* (the phylogenetically closest species to *Scorpaena porcus*), we performed a gene annotation using the BRAKER v2.1.6 pipeline with default parameters as input in its genome assembly deposited in GenBank (Accession: GCA_947000775.1) [35,36,37,38,39,40,41]. We then extracted the predicted proteins using the AGAT toolkit v.1.4.0 and employed the Orthofinder v2.5.4 software to identify the Piscidin-1 ortholog protein by providing a known set of teleost Piscidin-1 proteins from the Uniprot database (Accessions: A0A3Q8BH10, A0A7L7S7C3, and Q8UUG0) [42]. Finally, we functionally annotated the *P. miles* Piscidin-1 ortholog and the teleost Uniprot proteins with the addition of *Gadus morhua* Piscidin-1 (Accession: D4HRB8) through the InterProScan v100.0 online webserver [43].

### 2.4. Primary Antibody

An affinity-purified, rabbit-polyclonal, anti-Piscidin-1 antibody raised against the whole mature peptide sequence of Atlantic cod Piscidin-1 (GenScript, Piscataway, NJ, USA) was used in the present study. As reported by Ruangsri et al. [21], polyclonal antibodies were produced using the peptide antigen corresponding to C-FIHHIIGWISHGVRAIHR AIHG in accordance with the manufacturer’s instructions. In short, rabbits were given injections of the synthetic peptide after it had been conjugated to keyhole limpet hemocyanin (KLH). After that, the antiserum was affinity-purified by passing it over a column that included an immunosorbent made of cyanogen-bromide-activated agarose and a 23-mer Piscidin-1 fragment [21]. ELISA verified the affinity-purified antibody’s resultant titer, which was 1:64,000. Less than 1% cross-reactivity was observed in the ELISA for the peptide-specific antibody. According to GenScript, 1% cross-reactivity is 100 times more antibody than what is needed to produce the same optical density with free KLH, conjugated KLH, or a free peptide that shares fewer than three amino acids in the sequence. Moreover, the anti-Piscidin-1 antibody’s immunoreactivity was strong against 1 μg of Piscidin-1 peptide, but it did not react with synthetic Piscidin2 or Piscidin2b peptide (1 μg of Piscidin2; FLHHIVGLIHHGLSLFGDRAD or Piscidin2b; and FLHHIVGLIHHGKLDMYRSNN) from Atlantic cod, according to the Western blot analysis.

### 2.5. Immunoperoxidase

Piscidin-1 (GenScript, NJ, USA, dilution 1:100, source: rabbit) was assessed using an optical microscope and through immunohistochemical techniques. The slices were incubated for 60 min at room temperature with a goat anti-rabbit IgG-peroxidase conjugate (Sigma-Aldrich, St. Louis, MO, USA, dilution 1:100, source: goat) after being treated with the antibody for an entire night in a humidified environment. The sections were incubated for 1 to 5 min at room temperature in a solution containing 0.02% diaminobenzidine (DAB) and 0.015% hydrogen peroxide to measure the peroxidase activity of the sections. Afterwards, the slices were cleaned in PBS, dried, mounted, and examined with an Alexasoft TP3100A CMOS digital camera and a Zeiss Axioskop 2 plus microscope. The primary antibody was not used in a negative control trial. No counterstaining was used.

### 2.6. Immunofluorescence

After gradually deparaffinizing the slices, they were rehydrated in PBS and incubated for an hour in 2.5% bovine serum albumin (BSA). After that, the sections were left overnight at 4 °C in a humid environment and treated with a primary antibody against Piscidin-1 (GenScript, NJ, USA, dilution 1:100, source: rabbit). After exposing the primary antibody to it for one night, an Alexa Fluor 594 donkey anti-rabbit IgG TRITC conjugate (Molecular Probes, Invitrogen, Eugene, OR, USA, 1:300) was utilized. To prevent photobleaching, the sections were mounted using Fluoromount^TM^ (Diagnostic BioSystems Inc., Pleasanton, CA, USA). As a negative control, the experiments were conducted without the main antibody. Sections were analyzed, and images were taken using a Zeiss LSM DUO confocal laser scanning microscope equipped with a META module (Carl Zeiss MicroImaging GmbH, Jena, Germany). After each image was digitized, a 2048 × 2048-pixel array with 8-bit resolution was produced. Zen 2011 (LSM 700, v 3.0, Zeiss software, Oberkochen, Germany) was used to improve the photos. Each image was captured quickly to avoid photodegradation. To make the figure montage, digital photo cropping was performed using Adobe Photoshop 2023 (Adobe Systems, San Jose, CA, USA) [44].

## 3. Results

A total of 40 specimens (10 per maturity stage) were evaluated. The mean total length and the mean total weight of the analyzed specimens belonging to each macroscopic maturity stage are reported in Table 2. 

**Figure 1:**
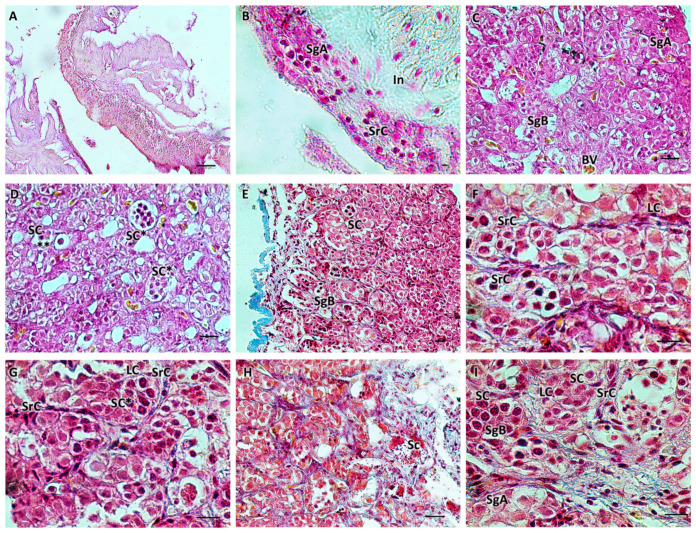
(**A**,**B**) Immature testis (M1), Mallory trichrome. The testis is composed of germinal and interstitial tissue. Spermatogonia and scattered Sertoli cells are visible. (**C**–**I**) Testis in early developing stage (M2), Mallory trichrome. Connectivity decreases, the number of spermatogonia A increases, and spermatogonia B appears with more densely stained nuclei. Juvenile spermatocysts containing 2 to 4 cells are visible. Some cysts appear at a more advanced stage of development with more than 4 cells (*). Sertoli cells surrounding the cysts are scattered; roughly polygonal Leydig cells are evident. B-spermatogonia (**). Primary and secondary spermatocytes begin to form. The organ appears to be rich in blood vessels. Legend: In = interstitium; SgA = spermatogonia A; SgB = spermatogonia B; SrC = Sertoli cells; LC = Leydig cells; Sc = spermatocytes; SC = sperm cysts; and BV = blood vessels. Magnification and scale bars: (**A**,**E**) 20×, 40 µm; (**B**–**D**,**H**) 40×, 20 µm; (**F**,**G**,**I**) 100×, 10 µm.

Histological examination of the testes showed the elongated shape of the organs, mainly composed of germinal and interstitial compartments separated by a basement membrane. The germinal compartment comprised the germinal epithelium, consisting of two types of cells: germ cells and somatic epithelial cells, specifically the Sertoli cells. In the immature gonads (M1), germ cells were found, single or in pairs, at the spermatogonial type A (SG A) stage, without connections through cytoplasmic bridges between Sertoli cells. Gonads with early-stage active spermatogenesis (M2) showed the presence of cysts containing B-type spermatogonia (SG B). Younger cysts initially contained two to four cells. The germinal cysts contained germ cells at different developmental stages. The *interstitium*, which fills the intertubular spaces, was mainly composed of fine collagenous connective tissue with scattered polygonal-shaped Leydig cells, a central nucleus, and blood capillaries. The immature stage (M1) to the developing virgin stage (M2) included spermatogonia A and B and spermatocytes. The spermatogonia of type A were rounded cells with large, rounded vesicular nuclei and a weakly stained cytoplasm. Spermatogonia type B were smaller in size, with dense chromatin clumps within their nuclei. Primary and secondary spermatocytes were smaller than spermatogonia, with spherical, large, and chromatin-rich nuclei (Figure 1).

**Figure 2:**
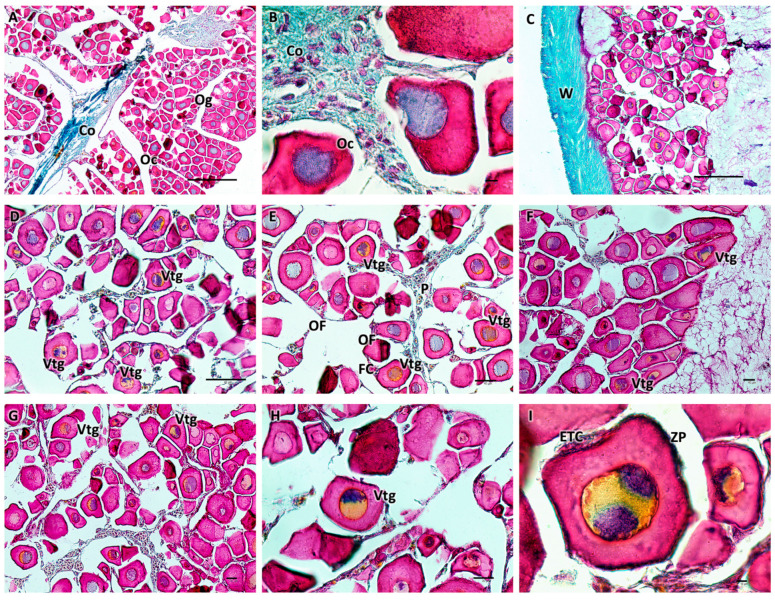
(**A**,**B**) Immature ovary (F1), Mallory trichrome. The ovary is cystic with numerous immature oogonia and oocytes and abundant connective tissue. At this stage, the nucleus is about half the size of the oocyte. (**C**–**I**) Ovary in early developing stage (F2), Mallory trichrome. There is a decrease in connective tissue, and some oocytes begin to enter the vitellogenesis stage, showing enlargement of the cytoplasm. The wall begins to thicken, which is thought to result in the formation of the zona pellucida. The oocytes are organized into ovarian follicles connected to the stroma by an epithelial peduncle. Legend: Og = oogonia; Oc = oocytes; Vtg = oocyte in early vitellogenesis; ZP = zona pellucida; ETC = external theca cells; OF = ovarian follicles; P = peduncle; FC = follicular cells; W = wall; and Co = connective tissue. Magnification and scale bars: (**A**,**C**) 10×, 50 µm; (**B**,**I**) 100×, 10 µm; (**D**–**G**) 20×, 40 µm; and (**H**) 40×, 20 µm.

Histological examination of the ovaries showed an ovarian wall consisting of three layers: the innermost layer was covered by a simple cuboidal epithelium; the middle layer had two sublayers (an outer one formed by a circular muscle system and an inner one formed by a much thicker, longitudinal muscle system) containing smooth muscle and numerous blood vessels; and an outer layer composed of mesothelium. The appearance of the ovary was cystic. In females, during the immature stage (F1), small polygonal oogonia and small oocytes were evident, and there was abundant fibrous matrix connective tissue. The nucleus, in the chromatin perinuclear stage, was about half the size of the oocyte. In the developing virgin maturity stage (F2), the connective tissue diminished, and the oocytes entered the early stages of vitellogenesis, with an enlargement of the cytoplasm. The wall of some oocytes began to thicken, which was likely a result of the formation of the zona pellucida. Ovarian follicles were connected to the stroma with a narrow, vascularized pedicle formed by a smooth, monostratified lamellar epithelium. The length of the pedicle increased as vitellogenesis progressed (Figure 2).

**Figure 3:**
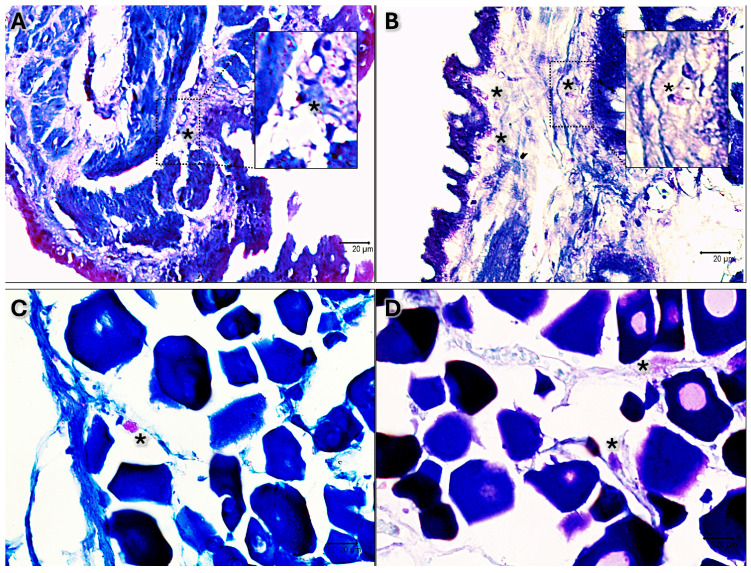
May–Grünwald Giemsa-stained sections 40×. (**A**) Immature testis. (**B**) Testis in early development. (**C**) Immature ovary. (**D**) Ovary in early development. Scattered immune cells are evident as pink-purple or blue in each stage of maturation of both male and female black scorpionfish gonads (*).

The presence of clear immune cells in both gonads at each stage of maturation is substantiated by May–Grünwald Giemsa histochemical staining, which is specifically employed for the identification of immune and blood cells (Figure 3).

**Figure 4:**
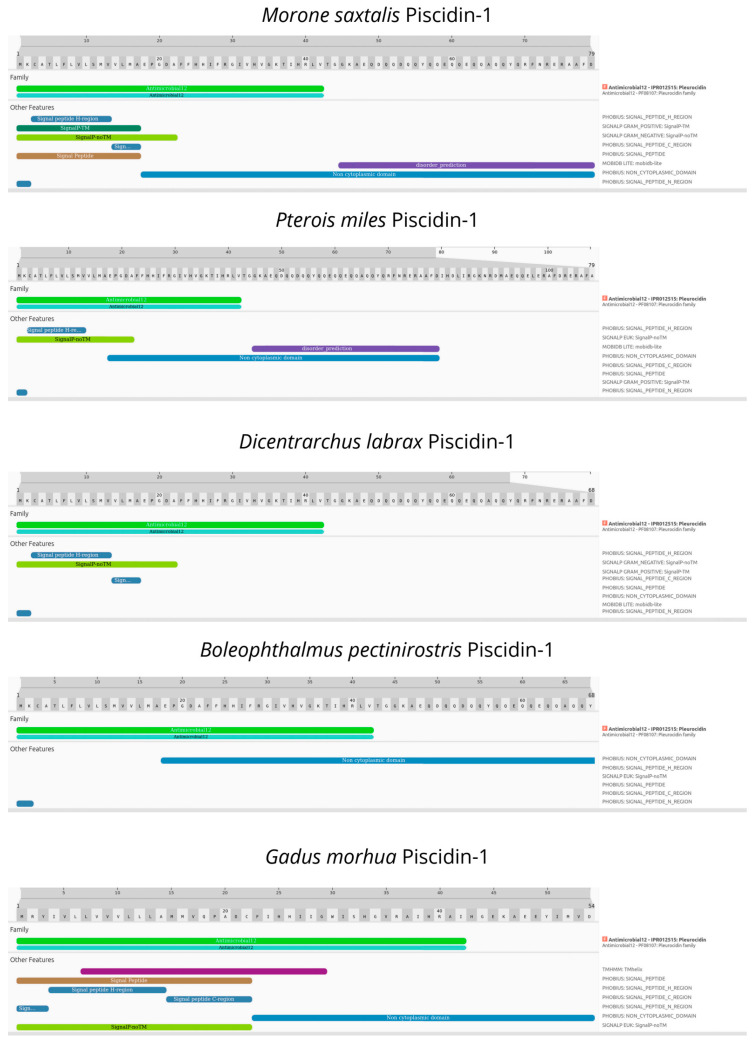
Structural and functional domains of Piscidin-1 among teleost obtained by InterPro Scan.

Our in silico analyses of Piscidin-1 revealed the presence of a Piscidin-1 protein ortholog in a close relative of the black scorpionfish, consisting of a 108 aa protein precursor. Moreover, the functional annotation analysis found the pleurocidin domain (IPR012515) characteristic of piscidins across all of the investigated teleost (Figure 4). 

**Figure 5:**
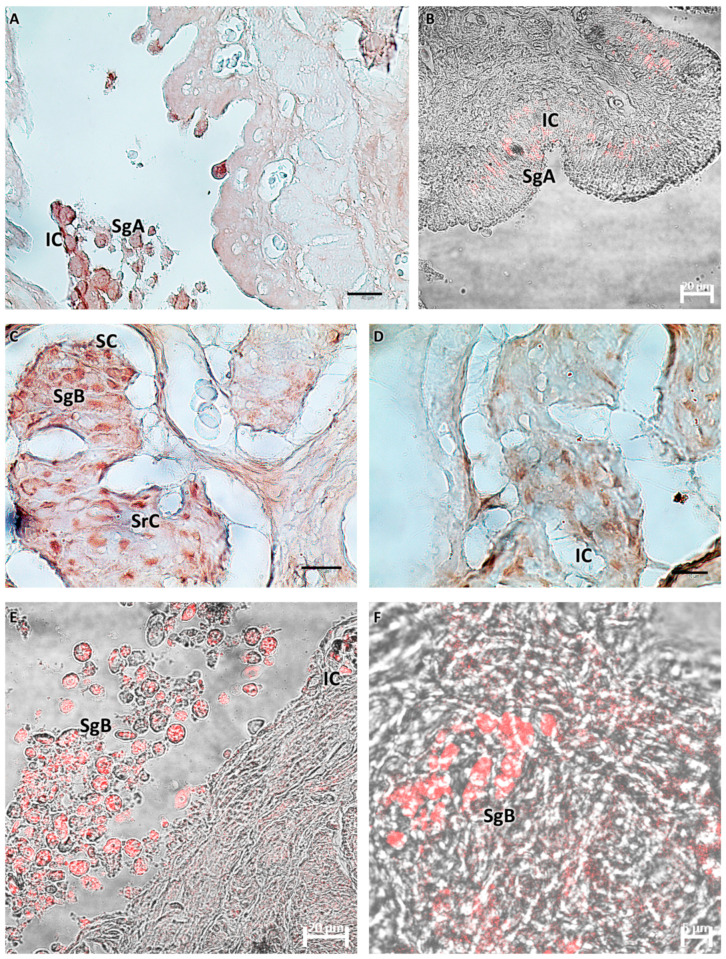
(**A**,**B**) Immature testis (M1), immunoperoxidase, and immunofluorescence. There is weak immunoreactivity of germ cells to Piscidin-1, especially spermatogonia A. There are a few scattered reactive immune cells. (**C**–**F**) Testis in early maturation (M2), immunoperoxidase and immunofluorescence. Spermatogonia B and spermatocytes appear positive for Piscidin-1, as do some Sertoli cells. Scattered immune cells appear strongly reactive to the antibody tested. Legend: SgA = spermatogonia A; SgB = spermatogonia B; SrC = Sertoli cells; SC = sperm cysts; and IC = immune cells.

**Figure 6:**
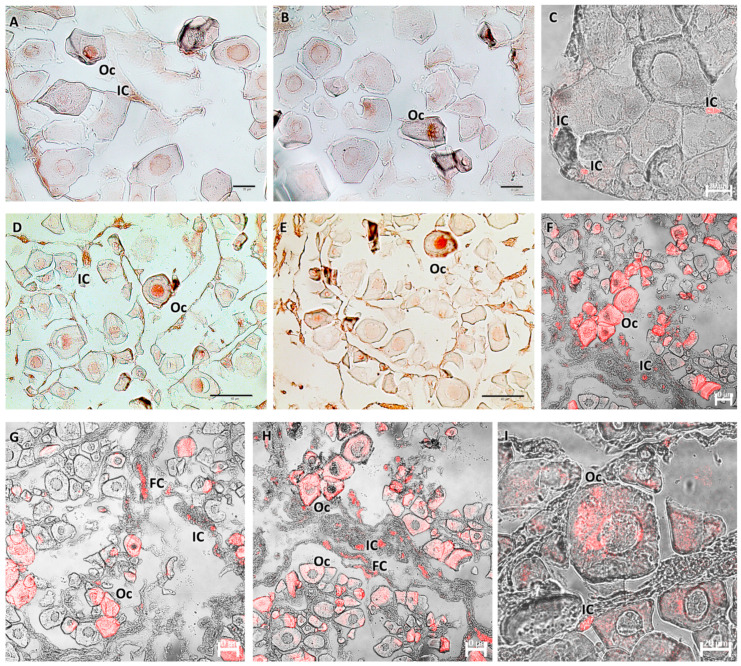
(**A**–**C**) Immature ovary (F1), immunoperoxidase, and immunofluorescence. Bland-positive oocytes and immune cells reactive to Piscidin-1 are visible. (**D**–**I**) Ovary in early developing stage (F2), immunoperoxidase and immunofluorescence. There is a more pronounced oocyte response to Piscidin-1, with reactivity involving the entire cell. Clearly visible and strongly labeled immune and follicular cells appear. Legend: Oc = oocytes; IC = immune cells; and FC = follicular cells.

At immature stages, in both male (M1) and female (F1) gonads, there was a low positive reaction to Piscidin-1 in reproductive cells. In contrast, immune cells appeared strongly immunoreactive to the antibody, more in females than in males. During the early stages of gonadal maturation, the testis (M2) exhibited a more pronounced spermatogonia immunoreactivity with strongly reactive spermatocytes (Figure 5). Similarly, in the ovary (F2), a considerable number of oocytes entered the vitellogenesis phase and showed immunoreactivity to Piscidin-1 (Figure 6). Additionally, both gonads exhibited positive somatic cells in the connective tissue, some Sertoli cells in the testis, and immune cells.

## 4. Discussion

The Scorpaenidae family comprises 219 species, of which only 17 have been identified in the last decade [45]. *S. porcus*, commonly known as the Black scorpionfish, is a small benthic fish with a sturdy body, typically measuring between 15 and 20 cm in total length. This species can be found in the Mediterranean and the Black Sea, as well as the eastern Atlantic regions of Morocco, the British Isles, the Azores, and the Canary Islands [23,46].

Fish testes are typically paired, elongated organs that are attached to the dorsal body wall by a fibrous sheath of connective tissue called the mesocortex. They are situated in the coelomic cavity, dorsal to the intestine, and ventral to the swim bladder. Each elongated testis has an efferent duct emerging from its meso/dorsal surface. It flows into the spermatic duct, forming a brief, unpaired segment before emerging into the urogenital papilla. The testicular duct system is formed by the efferent and spermatic ducts. It is responsible for transporting spermatozoa and is involved in sperm storage, nutrition, reabsorption, secretion of seminal fluid, and induction of sperm motility by changing the ionic composition of seminal fluid [47]. 

The histological analysis of *S. porcus* testis showed a decrease in connective tissue from the immature to the developing virgin stage. There were different numbers of spermatogonia A and B, and primary and secondary spermatocytes in the later stages. These findings are in line with a previous comparative study on *Lepisosteus osseus* (Linnaeus, 1758), *Scianops ocellatus* (Linnaeus, 1766), *Oreochromis mossambicus* (Peters, 1852), and *Centroponus undecimalis* (Lacepede, 1802) [48]. Especially during the M2 stage, Sertoli cells, which form cystic structures with germ cells, become clearly visible. Germ cell development begins with spermatogonial stem cells (SSCs), the most undifferentiated type of spermatogonial cells, which typically appear as single cells. When an SSC enters the differentiation pathway to become a spermatozoon, it exhibits a specific behavior during division in all animals. At the end of mitosis, when two new cells are formed, cell division is incomplete in differentiating cells because the daughter cells remain interconnected by a cytoplasmic bridge instead of forming two separate cells. These bridges also form during all subsequent germ cell divisions. An interconnected clone of cells is formed by germ cells generated from a specific SSC. All members of the same clone are synchronized in their activities through cytoplasmic bridges; they are at the same developmental stage and then disrupted when germ cells leave the germinal epithelium as sperm [47,49]. Furthermore, Leydig cells, which exhibit an organization typical of steroid hormone cells, were found in both analyzed stages. According to Marina et al. (2002), a study on the spotted ray, *Torpedo marmorata* (Risso, 1810) showed that the cells found in the gonad, including Sertoli cells, are highly involved in spermatogenesis and steroidogenesis, and are particularly active and present in mature spermatocysts [50].

Based on their structural characteristics, teleost ovaries can be divided into two primary categories: gymnovarian and cystovarian. It is crucial to understand that there are no functional distinctions between the two types of ovaries; rather, this classification is based only on anatomical variations. Oocytes are discharged directly into the coelomic cavity, exiting through the genital papilla in gymnovarian ovaries, which are open in the abdominal cavity and lack the ovarian duct. An ovarian membrane, which may or may not be muscular, encloses the ovary in cystovarian ovaries. Additionally, there is an ovarian duct that connects the oocytes to the external environment [51]. In the dorsal region of the peritoneal cavity, the ovaries of *S. porcus* are paired, saccular, and totally isolated from one another by uniting right before the genital opening. The ovary is categorized as a cystovarium by Porcu et al. (2022) [52]. The circulatory system passes via the ovary’s center, where the stroma grows radially around it. Every part of the ovary is surrounded by the ovarian cavity. Sahin et al. (2017) also reported that the core stroma is formed from connective tissue, which includes germ cells (oocytes) extending radially from the ovarian hilum to the ovarian wall surrounding it [53]. During the immature (F1) stage, the ovary contains polygonal and immature oocytes, which, during the F2 stage, enter the early vitellogenesis phase. A peduncle with extensive vascularization supplies the oocytes; it grows longer as the diameter of the cells increases. Specialization in this area is observed in both oviparous and viviparous animals, as well as in several other vertebrates, including birds and reptiles [54]. The structure serves numerous purposes. When mature oocytes ovulate into gelatinous masses, the peduncles of oviparous species—like those in the Scorpaena genus—avoid oocyte crowding [52]. 

In both male and female gonads, scattered immune cells have been identified by May–Grünwald Giemsa histochemical staining, which is a technique specific for immune cells, as reported in several studies [15,18,55,56,57,58]. The identification of immune cells is based on their morphology and location in the tissue, as supported by several studies [59,60,61]. Somatic cells are described and recognized based on their morphology and any communication with adjacent cells. The results presented here are in full agreement with those of previous articles, which similarly recognize and describe somatic cells by morphology [48,52,53].

Gonads are not immune-devoid organs. There is a strong connection between the defense and hormonal systems in fish gonads [53]. The interstitial tissue of the testis expresses both major histocompatibility complex (MHC) class I and II molecules. Furthermore, the vascular endothelium allows the trafficking of immune cells and immune effectors, such as cytokines, immunoglobulins, and complement proteins, into the testis [62]. The testicular interstitium of certain fish species contains both myeloid and lymphoid immune cell types. There have been reports of variations in leucocyte counts at various phases of reproduction. In the testicular region of the gonad of *Sparus aurata* (Linnaeus 1758), researchers have found acidophilic granulocytes, macrophages, B and T lymphocytes, and mast cells [63]. The genes for pro- and anti-inflammatory cytokines, chemokines, immunoglobulins, complement factors, antimicrobial peptides (AMPs), immune receptors, like Toll-like receptors (TLRs) and T-cell receptors (TCRs), and molecules involved in the antiviral response are expressed in the testes of sea bream and sea bass, similar to mammals [63]. 

Moreover, other fish species have gonad-specific molecules linked to immune–endocrine interactions. In female rainbow trout (*Oncorhynchus mykiss*, Walbaum, 1792), interferon (IFN)-3/4 is highly and specifically expressed in the ovary [64]. Two hepcidins have been described in the testis of the mudskipper (*Boleophthalmus pectinirostris*, Linnaeus, 1758) as gonad-specific AMPs. Hepcidin-2 is highly produced in Leydig cells, regulated by gonadotropins, and upregulated upon stimulation with lipopolysaccharide (LPS) [65]. Furthermore, a β-defensin that was expressed exclusively in the pituitary gland and testis of the orange-spotted grouper (*Epinephelus coioides*, Hamilton, 1822) was cloned [10]. Direct contact between leucocytes and the follicular wall, as well as between the steroidogenic theca and granulosa cells, has been seen in *Leucoraja erinacea* (Mitchill, 1825) [66]. In gilthead sea bream, acidophilic granulocytes are present in large numbers during larval development in the ovarian area and remain until they are fully developed [61]. They are restricted to the testicular interstitium and the connective tissue surrounding the ovarian and testicular regions in mature gonads [61]. 

In this study, we detected Piscidin-1-immunoreactive immune cells in the connective tissue of both male and female gonads during early sexual maturation in *S. porcus*. Furthermore, we observed reactivity to Piscidin-1 in several Sertoli cells and oocytes during early vitellogenesis. These new data are consistent with previous research on other fish species [61,67,68,69,70,71]. Piscidin-1 is constitutively present in immune-related organs of naïve fish, according to a study on Atlantic cod by Ruangsri et al. (2012), indicating a major role for this peptide in the innate immune system of Atlantic cod. Furthermore, Piscidin-1 has been found in a few nonimmune tissues and organs, indicating additional possible functions for this widely distributed peptide in preserving homeostasis in Atlantic cod [21]. Valero et al. (2015, 2018) demonstrated the high expression of several AMPs, including piscidin, in the male gonads of *Dicentrarchus labrax* (Linnaeus, 1758), suggesting their possible involvement in vertical immune transmission [72,73].

Our findings underscore the significance of cross-talk between the immune and reproductive systems. Several studies have highlighted the link between immune response and some aspects of fish ecology and reproduction. Specifically, certain AMPs are locally regulated by gonads, influencing fish physiology and behavior [72]. It has been demonstrated that immunocompromised male fish reduce mating attempts and are more readily avoided by females [74]. Given that fish reproduction is seasonal, their immune systems are frequently exposed to hormonal changes, exhibiting immunological strategies that can shift from reproductive to nonreproductive periods [53,75]. 

## 5. Conclusions

In conclusion, our data provide information on the early stages of the reproductive development of the gonads in Black scorpionfish. These results are potentially useful for better understanding the species’ reproductive cycle. Improving the knowledge base on the reproductive biology of highly commercially exploited species is essential for conservative purposes, as this information is important to enhance their management considering the ever-growing fishing pressure affecting the demersal and coastal fishery resources. Moreover, the novel results obtained regarding the expression of the AMP Piscidin-1 contribute to our basic knowledge of the defense system of this teleost and its interaction with reproduction, which is a key event for the continuation of the species.

## Figures and Tables

**Table 1:** Macroscopic criteria for interpretation of gonadal stages of development.

	Macroscopic Criteria
M1	The testis is smaller than a third of the body cavity and is slender and white in color.
M2	The testes, thin and white, occupy less than half the body cavity until they become roughly symmetrical, pinkish in color, and measure about half the length of the body cavity.
F1	The tiny, translucent, pinkish ovary is shorter than half of the body cavity. The naked eye is unable to see the eggs.
F2	The ovary is small, pinkish/reddish, and even translucent, occupying approximately half of the body cavity. Blood vessels are visible, but the eggs are not visible to the naked eye.

**Table 2:** Minimum (Min), maximum (Max), mean, and standard deviation (SD) of the total length (TL) and weight (TW) of the analyzed specimens (N = 40) for each macroscopically detected maturity stage.

	TL (mm)				TW (g)			
	Min	Max	Mean	SD	Min	Max	Mean	SD
Male specimens in the M1 stage	70	85	78.75	6.29	6.40	12.20	10.04	2.62
Male specimens in the M2 stage	80	165	130	30.09	9.63	93.4	50.17	29.88
Female specimens in the F1 stage	65	93	80	7.07	7.21	15.51	10.81	1.81
Female specimens in the F2 stage	70	230	143.93	44.06	8.17	230.5	75.96	64.61

## Data Availability

The original contributions presented in this study are included in this article; further inquiries can be directed to the corresponding authors.
